# Influence of Finish Line and CAM Unit on the Marginal Adaptation and Margin Trueness of Lithium Disilicate Glass‐Ceramic and Composite Resin Onlay Restorations

**DOI:** 10.1111/jerd.70087

**Published:** 2025-12-25

**Authors:** João Pitta, Gülce Çakmak, Maurice Salem, Philippe Mojon, Irena Sailer, Philippe Boitelle

**Affiliations:** ^1^ Division of Fixed Prosthodontics and Biomaterials University Clinics of Dental Medicine, University of Geneva Geneva Switzerland; ^2^ Department of Reconstructive Dentistry and Gerodontology School of Dental Medicine, University of Bern Bern Switzerland; ^3^ Department of Prosthodontics, Geriatric Dentistry and Craniomandibular Disorders Charité‐Universitätsmedizin Berlin, Corporate Member of Freie Universität Berlin and Humboldt‐Universität Zu Berlin Berlin Germany; ^4^ Department of Prosthodontics, Faculty of Dentistry Biruni University Istanbul Turkey; ^5^ Prosthodontic Department Faculty of Dentistry, Lille University, Lille University Medical Center Lille France

**Keywords:** bevel, butt joint, CAM unit, chamfer, composite resin, lithium disilicate, onlay

## Abstract

**Objective:**

To evaluate the marginal adaptation and margin trueness of lithium disilicate glass‐ceramic (LDS) and glass‐filler reinforced composite resin (CR) onlays fabricated using three different finish lines and two chairside CAM units (4‐ and 5‐axis).

**Methods:**

LDS (e.max CAD) and CR (Tetric CAD) onlays were fabricated on butt joint, chamfer, and bevel finish lines using 4‐axis (CEREC MC XL) and 5‐axis (Programill One) chairside CAM units (*N* = 120). Onlays were scanned for margin trueness and marginal adaptation analyses. Data were analyzed with three‐way ANOVA and Tukey B post hoc test (*α* = 0.05).

**Results:**

A significant interaction was found among finish line, material, and CAM unit for marginal adaptation and margin trueness (*p* < 0.001). Bevel had the highest marginal adaptation; chamfer the lowest. Butt joint showed the highest margin trueness; bevel the lowest. CR onlays had better marginal adaptation than LDS with bevel finish lines, regardless of CAM unit. Overall, 5‐axis CAM units yielded better margin trueness.

**Conclusions:**

Finish line design, restorative material, and CAM unit affected the marginal adaptation and margin trueness. The bevel finish line yielded the highest marginal adaptation and the chamfer the lowest, regardless of material or CAM unit. Conversely, the butt joint showed the highest margin trueness, and the bevel the lowest.

**Clinical Significance:**

Finish line design, material type, and CAM unit selection may affect marginal adaptation and margin trueness of onlays. A bevel finish line may improve marginal adaptation, while a butt joint finish line may enhance margin trueness. Using a 5‐axis CAM unit can improve trueness, particularly with bevel and chamfer finish lines and composite resin materials.

## 1 Introduction

Minimally invasive procedures using computer‐aided design and computer‐aided manufacturing (CAD‐CAM) are a key aspect of modern prosthetic and reconstructive dentistry, shifting treatment from conventional full‐coverage crowns toward defect‐oriented partial‐coverage restorations (PCRs) [1, 2, 3]. Onlays are commonly used as PCRs to restore one or more cusps and adjoining occlusal surfaces while preserving healthy tooth structure [4, 5, 6, 7]. The long‐term success of PCRs depends on factors like material loss, tooth topography, preparation design, finish line, adhesive protocol, digital and fabrication methods, and material choice [4, 5, 6, 7, 8, 9].

For PCR fabrication, glass‐ceramics and composite resins (CRs) are commonly used, each with distinct advantages and disadvantages [5, 6, 7, 10, 11, 12, 13, 14, 15, 16, 17]. Glass‐ceramics are preferred when esthetics are crucial due to superior optical and surface properties, plus high compressive strength, wear resistance, and chemical durability [10, 13, 14]. CRs are chosen for their ease of fabrication (easier milling, no firing required), intraoral repairability, reduced antagonist wear, shock absorption, and cost‐effectiveness [14, 15, 16, 17]. Different systematic reviews and meta‐analyses have reported the survival rates of onlays or PCRs as 93.7% for lithium disilicate glass‐ceramic (LDS) after 3 years [6], and 90% for CR and 98% for LDS over longer periods [17].

As the main advantage of PCRs lies in their minimally invasive approach enabled by improvements in adhesive techniques, determining the optimal preparation and finish line design is crucial for preserving intact tooth structure while ensuring restoration stability [18, 19]. Clinicians select finish line configurations such as chamfer, shoulder, beveled margin, or butt joint based on clinical scenarios and the properties of the restorative material properties [18, 19, 20, 21, 22, 23].

Marginal adaptation is critical for restoration success, as poor adaptation can result in microleakage, secondary caries, pulpitis, and periodontal inflammation, leading to clinical failure [18, 19, 20, 21, 22, 24, 25]. Fabrication accuracy is a key factor affecting marginal and internal adaptation [24, 25, 26, 27, 28]. Studies highlight concerns about finish line configurations due to digital scanning inaccuracies, milling errors, and marginal chipping, particularly with ceramics. These studies emphasize that the intrinsic properties of restorative materials affect survival, finish line design, fabrication accuracy, and marginal adaptation because of differences in mechanical behavior, milling characteristics, and clinical handling requirements [4, 6, 21, 22, 23, 28]. A recent systematic review found that CAM parameters—including the unit used, milling axes (3‐, 4‐, 5‐axis), and bur specifications—affect restoration trueness and adaptation [29]. Understanding these material‐ and system‐specific characteristics is essential for selecting appropriate finish lines and CAM units [29].

To the authors' knowledge, limited information exists regarding margin accuracy and adaptation of ceramic and CR onlays fabricated with different finish lines using different chairside CAM units [28]. Given the increasing use of 4‐axis chairside CAM units for onlay fabrication, comparing them with newer 5‐axis chairside CAM units is clinically relevant. Hence, the aim of the present study was to evaluate the marginal adaptation and margin trueness of LDS and CR onlays when fabricated on three different finish lines (butt joint, chamfer, bevel), using two chairside CAM units with different numbers of axes (4‐ and 5‐axis). The research hypotheses were that (i) the finish line, (ii) restorative material, and (iii) CAM unit would not influence the marginal adaptation and margin trueness of onlay restorations.

## 2 Materials and Methods

**FIGURE 1:**
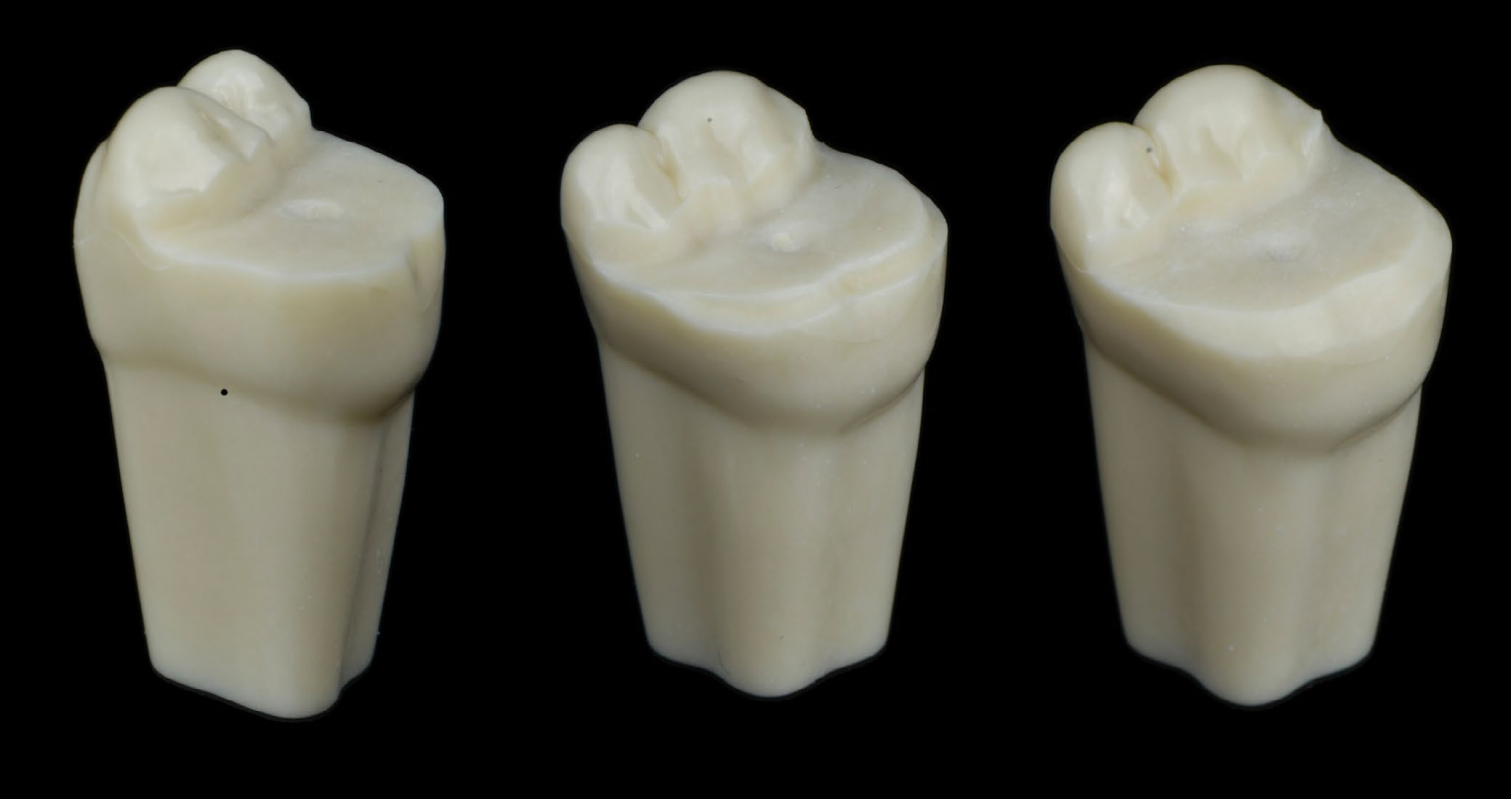
Representative images of onlay preparations using three different finish lines: Butt joint, chamfer, and bevel, respectively.

**FIGURE 2:**
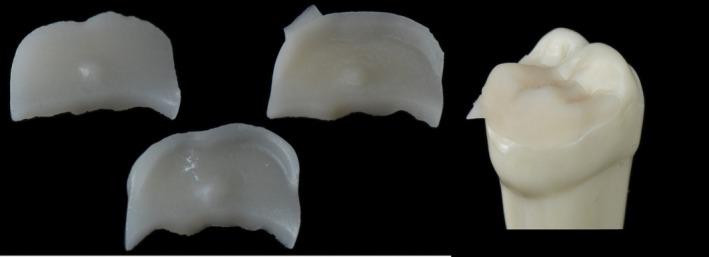
Representative images of fabricated onlay restorations for three different finish lines: Butt joint, chamfer, and bevel, and onlay seated on abutment tooth.

Three intact resin maxillary right first molars (ANA‐4 ZE; Frasaco GmbH) were prepared by one operator (P.B.) using three different finish lines (butt joint, chamfer (0.8 mm), and bevel vestibular finish line) for indirect onlay restoration (Figure 1). The mesio‐vestibular‐distal cavity preparation included mesial and distal cusp reduction using cylindrical and football‐shape diamond instruments (Intensiv Guided Universal Prep Set ref. 158; Intensiv SA, Montagnola Switzerland). After preparation, teeth were scanned using a laboratory scanner (inEos X5; Dentsply Sirona, York, USA) and 3 virtual models were generated by CAD software (InLab CAD Software; Dentsply Sirona, York, USA). Restorations were designed using manufacturer parameters: 500 μm minimum radial thickness, 2 mm minimum occlusal thickness, 50 μm die spacer, 20 μm marginal adhesive discrepancy, and 50 μm margin thickness. These designs were exported as reference onlay CAD standard tessellation language files (RO‐STLs) for restoration fabrication and margin trueness analysis. For each finish line, restorations were milled from LDS (e.max CAD; Ivoclar Vivadent AG, Schaan, Liechtenstein) or glass‐filler reinforced CR (Tetric CAD; Ivoclar Vivadent AG, Schaan, Liechtenstein) using two chairside CAM units: a 4‐axis (CEREC MC XL; Dentsply Sirona, York, USA) and a 5‐axis (Programill One; Ivoclar Vivadent, Ivoclar Vivadent AG, Schaan, Liechtenstein) CAM unit. A total of 120 onlays (*n* = 10) were fabricated (Figure 2). After milling, LDS onlays were crystallized according to manufacturer instructions (Programat P500; Ivoclar Vivadent, Ivoclar Vivadent AG, Schaan, Liechtenstein). No mechanical polishing or additional surface treatments were applied.

For margin trueness evaluation, fabricated onlays' internal and external surfaces were scanned using an optical laboratory scanner (Iscan D104i; Imetric 3D SA, Courgenay, Switzerland) (test onlay scan STL, TO‐STL). For marginal adaptation evaluation, a previously described triple scan method was applied [24, 30, 31]. Two additional scans were made using the same optical laboratory scanner: a prepared abutment teeth scan (master teeth scan) and an onlay seated on the abutment teeth scan (assembly scan). For the assembly scan, onlays were seated on prepared abutment teeth without resin cement. The optical laboratory scanner accuracy was 4 μm (manufacturer reported). Reverse engineering software (GOM Inspect 2019; GOM GmbH, Braunschweig, Germany) was used for margin trueness and marginal adaptation evaluation.

**FIGURE 3:**
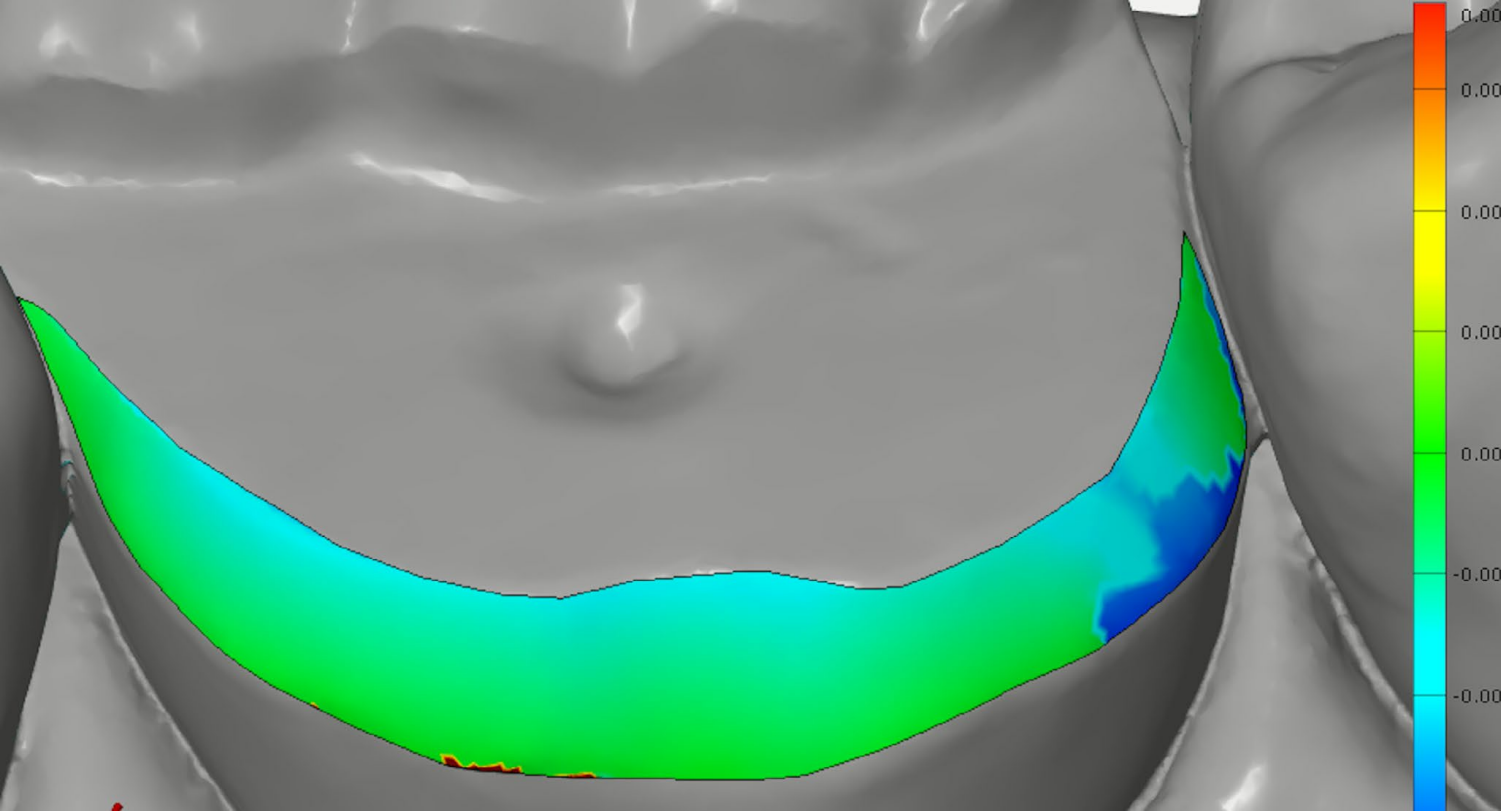
Representative image of 3D marginal adaptation analysis (internal space between onlay and marginal preparation surfaces).

For marginal adaptation evaluation, internal space between onlay and marginal preparation surfaces was measured using a triple scan method with equidistant surface points (GOM Inspect 2019; GOM GmbH, Braunschweig, Germany) [25]. First, the master teeth scan was imported and marginal areas were virtually separated using the “surface curve” function. Marginal areas were determined by two curves: the outer curve at the cavosurface angle and inner curve at the finish line where the axial wall begins. A surface patch was created for the marginal area on the master teeth scan (Figure 3) [25]. This was repeated for each abutment tooth, saved as a template for standardized analysis. Second, the assembly scan was imported and pre‐aligned to the master teeth scan. After pre‐alignment, surfaces excluding preparation areas were selected on the master teeth scan and section‐based “best fit alignment” was applied. Third, the test onlay scan was imported and aligned to the assembly scan using pre‐alignment, followed by section‐based “best fit alignment” using external onlay surfaces [25]. Marginal adaptation was measured at multiple points using the “comparison surface” function as previously described [25]. Measurements were averaged for each onlay.

**FIGURE 4:**
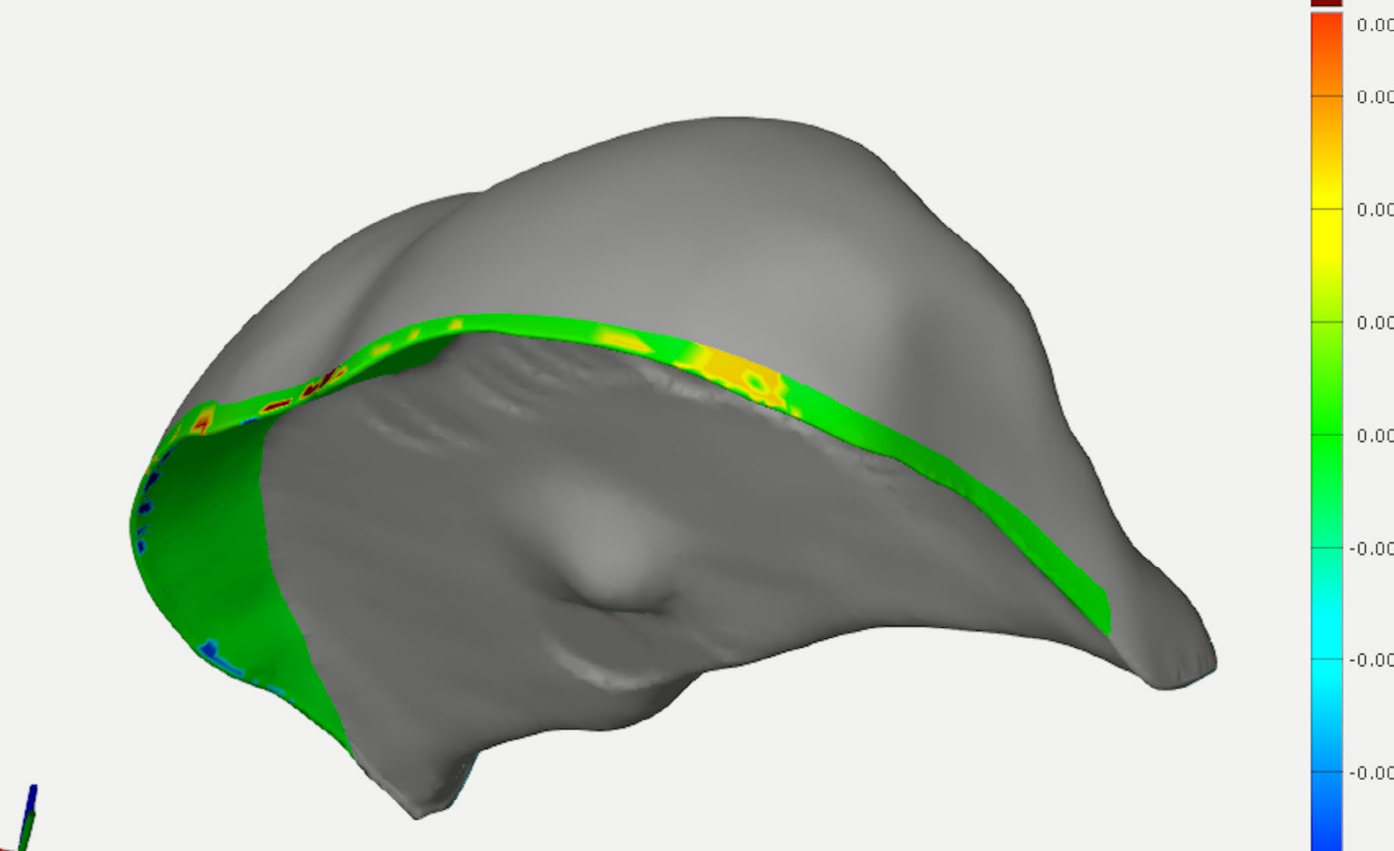
Representative image of margin trueness analysis (marginal area on reference onlay CAD standard tessellation language file (RO‐STL)).

For the evaluation of margin trueness, RO‐STLs were imported. The margin areas were segmented as described above using the “surface curve” function of the inspection software. The surface curve was positioned at 2 mm of the finish line, side intrados, and side extrados (Figure 4). A surface patch was created for the marginal area on the RO‐STL. Then, the TO‐STL was imported and pre‐aligned to the corresponding RO‐STL. The surface patches were selected to measure the accuracy with the “comparison surface” function. The mean values were used for the trueness.

A general linear model using median measurements as the dependent variable and finish line design (butt‐joint, chamfer, and bevel), restorative material (LDS and CR), and chairside CAM units (4‐ and 5‐axes) as fixed effect factors was conducted. The normality of the data distribution was verified by the Kolmogorov–Smirnov and Shapiro–Wilk tests. Three‐way analysis of variance (ANOVA) was used to analyze main effects (finish line, material, and chairside CAM unit) and interactions. The Tukey B post hoc test was used to identify significant differences between groups. Independent *t*‐tests were used to analyze differences between the CAM unit and material within each finish line design. Pearson correlation analysis was performed to evaluate the relationship between marginal adaptation and margin trueness. A statistical software program (IBM SPSS Statistics v24.0; IBM Corp) was used with a significance level of *α* = 0.05.

## 3 Results

The three‐way ANOVA (Table 1) revealed that finish line (*p* < 0.001), material (*p* < 0.001), and CAM unit (*p* < 0.001) had a significant effect on the marginal adaptation of onlays, with a significant interaction among them (*p* < 0.001). Tukey B post hoc test indicated significant differences among finish lines: bevel had the highest and chamfer the lowest marginal adaptation (*p* < 0.05), regardless of material and CAM unit (Table 2). With bevel, CR had higher marginal adaptation than LDS (*p* < 0.05), regardless of CAM unit. For chamfer, both materials showed similar adaptation regardless of CAM unit. For butt joint, LDS outperformed CR with the 4‐axis CAM unit, opposite to the 5‐axis CAM unit. When chamfer or bevel finish lines were used, 4‐ and 5‐axis CAM units resulted in similar marginal adaptation within both materials. LDS showed better marginal adaptation with butt joint using a 4‐axis CAM unit, whereas CR performed better with a 5‐axis CAM unit.

**TABLE 1:** Summary of ANOVA data for marginal adaptation.

Effect	Df	F ratio	*p*
Finish line	2	184.188	< 0.001
Material	1	31.644	< 0.001
CAM unit	1	33.280	< 0.001
Finish line × Material	2	20.302	< 0.001
Finish line × CAM unit	2	20.534	< 0.001
Material × CAM unit	1	91.648	< 0.001
Finish line × Material × CAM unit	2	74.588	< 0.001

Abbreviations: CAM, computer‐aided manufacturing; Df, numerator degrees of freedom.

**TABLE 2:** Descriptive statistics of marginal adaptation (mean distance, mm) for each restorative material‐CAM unit‐finish line pair.

Restorative material	CAM unit	Finish line	Mean	SD	Total mean	Total SD
LDS	4‐axis milling unit	Bevel	0.62^CD^	0.03	0.65	0.07
		Butt joint	0.59^bc^	0.02		
		Chamfer	0.75^E^	0.01		
	5‐axis milling unit	Bevel	0.66^D^	0.06	0.77	0.12
		Butt joint	0.91^F^	0.06		
		Chamfer	0.74^E^	0.02		
CR	4‐axis milling unit	Bevel	0.55^AB^	0.05	0.68	0.10
		Butt joint	0.75^E^	0.02		
		Chamfer	0.76^E^	0.02		
	5‐axis milling unit	Bevel	0.53^A^	0.02	0.65	0.11
		Butt joint	0.66^D^	0.07		
		Chamfer	0.78^E^	0.02		

*Note*: LDS, lithium disilicate glass‐ceramic (e.max CAD); CR, glass‐filler reinforced composite resin (Tetric CAD); 4‐axis CAM unit (CEREC MC XL); 5‐axis CAM unit (Programill One). Different superscript uppercase letters show significant differences.

Abbreviations: CAM, Computer‐aided manufacturing; SD, Standard deviation.

The 3‐way ANOVA (Table 3) revealed that finish line (*p* < 0.001), material (*p* < 0.001), and CAM unit (*p* < 0.001) significantly affected margin trueness, with a significant interaction among the factors (*p* = 0.029). The Tukey B post hoc test (Table 4) showed significant differences among finish lines. Butt joint had the highest margin trueness (*p* < 0.05) and bevel the lowest (*p* < 0.05), regardless of material and CAM unit. With bevel, CR and LDS had similar margin trueness. For chamfer, LDS showed higher trueness than CR with the 5‐axis CAM unit. For butt joint, LDS had higher trueness than CR with the 4‐axis CAM unit. Within LDS, 4‐ and 5‐axis CAM units resulted in similar trueness for butt joint. However, LDS had higher margin trueness when the bevel and chamfer finish lines were fabricated using the 5‐axis CAM unit. CR had higher margin trueness when the 5‐axis CAM unit was used regardless of finish line.

**TABLE 3:** Summary of ANOVA data for margin trueness.

Effect	Df	F ratio	*p*
Finish line	2	51.247	< 0.001
Material	1	38.403	< 0.001
CAM unit	1	269.275	< 0.001
Finish line × Material	2	6.594	0.002
Finish line × CAM unit	2	15.665	< 0.001
Material × CAM unit	1	7.325	0.008
Finish line × Material × CAM unit	2	3.672	0.029

Abbreviations: CAM, computer‐aided manufacturing; Df, numerator degrees of freedom.

**TABLE 4:** Descriptive statistics of margin trueness (mean distance, μm) for each restorative material‐CAM unit‐finish line pair.

Restorative material	CAM unit	Finish line	Mean	SD	Total mean	Total SD
LDS	4‐axis milling unit	Bevel	70^DE^	6	0.05	0.02
		Butt joint	18^AB^	13		
		Chamfer	49^C^	10		
	5‐axis milling unit	Bevel	28^B^	3	0.02	0.01
		Butt joint	11^A^	9		
		Chamfer	12^A^	5		
CR	4‐axis milling unit	Bevel	79^E^	17	0.07	0.02
		Butt joint	51^C^	13		
		Chamfer	64^CD^	11		
	5‐axis milling unit	Bevel	25^AB^	6	0.02	0.01
		Butt joint	19^AB^	17		
		Chamfer	29^B^	14		

*Note*: LDS, lithium disilicate glass‐ceramic (e.max CAD); CR, glass‐filler reinforced composite resin (Tetric CAD); 4‐axis milling unit (CEREC MC XL); 5‐axis milling unit (Programill One). Different superscript uppercase letters show significant differences.

Abbreviations: CAM, Computer‐aided manufacturing; SD, Standard deviation.

A weak but significant negative correlation (*r* = −0.271, *p* = 0.005) was found between margin trueness and marginal adaptation.

## 4 Discussion

Based on the findings of the present study, the finish line design, restorative material, and CAM unit significantly affected both marginal adaptation and margin trueness of onlay restorations. Therefore, the null hypotheses were rejected.

The performance of a manufacturing process can be described in terms of accuracy, which encompasses both trueness (the closeness of the average of a set of measurements to the true value) and precision (the closeness of repeated measurements to each other) [24]. Marginal adaptation reflects the overall accuracy of the complete CAD/CAM workflow—including data acquisition, design, manufacturing, and milling—whereas margin trueness isolates the fidelity of the manufacturing and milling stages specifically [24]. Poor trueness does not indicate an inadequate design but rather a dispersion or distortion of data that occurs within or between the manufacturing and milling steps [24]. This study results showed that CR achieved superior marginal adaptation with a bevel finish line, regardless of the CAM unit used. Furthermore, it outperformed LDS under the same conditions. This may be attributed to the relatively ductile nature of CR, along with its inherent flexibility, lower modulus of elasticity, and lower hardness value. These properties reduce the risk of fracture and chipping, improve machinability, and may allow better adaptation to the angled margins of the bevel [6, 22, 24]. These findings are in line with the results of a previous study, which reported that the marginal gaps of CAD‐CAM inlays varied depending on the material used, with CR inlays showing smaller marginal gaps than LDS inlays [26]. Similarly, CR crowns were found to have lower marginal gap values compared to LDS crowns [27]. Supporting these findings, Pilecco et al. [32] reported that the same 4‐ and 5‐axis machines produced different marginal, cervical, and pulpal chamber fits in endocrowns. CR (Tetric CAD) showed smoother margins and better marginal fit than LDS (IPS e.max CAD), likely because of its flexibility and homogeneous structure, which may enhance its adaptation to complex geometries [32]. Therefore, when CR is selected as the restorative material, a bevel finish line should be preferred to have better marginal adaptation. However, LDS and CR had similar marginal adaptation with the chamfer finish line. This finding highlights the importance of selecting the appropriate finish line based on the restorative material used.

LDS generally showed slightly better marginal trueness, particularly when chamfer and butt joint finish lines were used in combination with certain CAM units. Although the mean discrepancies in margin trueness ranged from 8 to 33 μm, such differences can still be clinically relevant, considering that marginal gaps of up to 100 and 120 μm are typically regarded as clinically acceptable. Gaps smaller than 80 μm are undetectable by probing; however, a minimum space of approximately 30 μm is necessary to ensure adequate cement space and bonding integrity. Hence, small variations in trueness can directly determine whether a restoration falls within clinically acceptable limits for marginal gaps. In contrast to trueness, LDS demonstrated superior marginal adaptation when used with a bevel finish line. Although LDS is a brittle material with a rigid crystalline structure, the smoother and more continuous surface produced by a bevel may facilitate more precise milling and easier seating, reducing the likelihood of premature contacts that prevent full seating and increase marginal discrepancies. Therefore, a bevel finish line can be recommended regardless of the CAM unit used, whereas a butt joint finish line is more suitable for 4‐axis units [7, 13].

Regardless of the material and CAM unit used, the bevel finish line showed the highest marginal adaptation but the lowest margin trueness, while the butt joint finish line demonstrated the highest margin trueness and the chamfer finish line the lowest marginal adaptation. In addition, there was a negative correlation between margin trueness and marginal adaptation. The contrasting results in margin trueness and marginal adaptation may be explained by the distinct geometric features of each finish line design. The bevel finish line, with its angled surface, may complicate accurate scanning and milling, reducing margin trueness. However, its design can provide closer seating of the restoration, enhancing marginal adaptation. In contrast, the butt joint finish line is a flat, well‐defined margin that might be easier to scan and mill accurately, resulting in higher margin trueness. However, this design may not provide a clear and stable seating position as closely as the bevel [19, 22], potentially compromising marginal adaptation. The chamfer finish line is characterized by a curved, sloped margin that might be more difficult to scan and mill, potentially leading to lower marginal adaptation and reduced trueness. This is consistent with existing literature, which reports that traditional designs incorporating beveled margins yield smaller marginal and internal discrepancies for CR onlays than shoulder preparations [22]. Although the shoulder finish line was easier to prepare, scan, and fabricate, it resulted in greater marginal and internal discrepancies compared to the traditional design [14]. Similarly, Lima et al. [21] reported that preparation design influences the marginal adaptation of nanoceramic resin onlays. Specifically, onlay preparations with a modified shoulder margin showed better marginal adaptation compared to those with flat cuspal reduction without a defined shoulder margin [21]. Moreover, a systematic review has shown that the planned cement space may not always be accurately reproduced, as it depends on the milling parameters. This can affect the adaptation of the restoration at various points [29]. Therefore, beyond margin trueness, the trueness of the inner surface of the restoration, as well as the presence of over‐ or undercontoured areas, may significantly influence the seating and overall fit of the restoration. This may help explain the inconsistency observed between trueness and margin adaptation results.

One of the main disadvantages of milling technology is the difficulty in fabricating complex preparation designs, as CAM units are unable to mill contours in areas smaller than the diameter of the burs [29]. Previous studies have recommended avoiding sharp angles to allow adequate bur access, while favoring smooth, flat surfaces, fewer retentive features, and divergent walls for better milling efficiency and accuracy [29, 33, 34]. A recent scoping review [29] found that variations in bur types, milling modes, and the number of bur uses affected internal adaptation and surface trueness of milled indirect restorations [29]. Restorations produced by 5‐axis units generally demonstrated better adaptation than those made with 3‐ and 4‐axis units due to enabling rotation of the block in additional A‐axis and C‐axis and allowing milling steep walls, small angles, and undercuts [4, 29]. In the present study, the 5‐axis unit showed higher margin trueness for LDS with bevel and chamfer finish lines, and for CR regardless of the finish line, highlighting its superior ability to mill intricate margins more accurately. However, both the 4‐ and 5‐axis CAM units produced similar marginal adaptation for both materials when chamfer or bevel finish lines were used, as well as similar margin trueness for LDS when the butt joint finish line was applied. Hence, it could be interpreted that finish line selection should align with the material selection and CAM unit selection should be considered as an additional factor [28].

Previous studies suggest that marginal gaps up to 100 and 120 μm have been considered clinically acceptable; however, smaller gaps may yield superior outcomes in terms of restoration fit [4, 20, 30]. In the present study, all combinations of finish line design, material, and CAM unit resulted in marginal gaps that exceeded the clinically acceptable threshold of 120 μm [28]. Previous studies have also indicated that the marginal adaptation of CAD‐CAM onlays is influenced by the method of evaluation [31, 35]. One possible explanation for the high marginal gap outcome can be the scanning protocol used in the triple‐scan method. Unlike some previous studies [24, 28], that applied a standardized pressure of 2 N during scanning, no additional pressure or cement was used in this study during scanning. This may have contributed to the larger marginal gaps observed between the abutment teeth and the restorations. Although the triple‐scan protocol is considered reliable, nondestructive, and straightforward, its accuracy and reproducibility have been reported to depend on scan quality and proper alignment of the 3D datasets [24, 28, 31]. Therefore, the findings should be interpreted with caution, as different results might be obtained using alternative measurement methods such as microcomputed tomography [11], the silicone replica technique [22], stereomicroscopy, optical microscopy, direct viewing, cross‐sectioning methods [35], or scanning electron microscope.

One limitation of the present study is the use of a single type of CAM technology. Future studies should evaluate variations in bur types, milling modes, number of bur uses, and cement space settings. With the increasing adoption of 3D‐printing technology and resin composite onlays [24, 28], it is also important to investigate different 3D‐printed composites, as well as various milled glass‐ceramics and composite resins. Another limitation is that, although resin cement, its viscosity, and the cementation technique can influence onlay adaptation, these factors were not evaluated in this study. In addition, different scanners and cement space settings might yield different outcomes [4]. The use of LDS introduces crystallization as a factor that may affect marginal accuracy due to stress from firing and cooling, which warrants investigation in future studies [7]. As reported, the marginal adaptation of LDS and CRs is acceptable in the short and mid‐term, while deterioration becomes evident in the long term [6, 36, 37]; therefore, future clinical studies are needed to validate the present findings.

## 5 Conclusion

Within the limitations of this current study, it was concluded that:

1. Finish line design, restorative material, and CAM unit significantly influenced both margin trueness and marginal adaptation of onlays.
2. The bevel finish line yielded the highest marginal adaptation and the chamfer the lowest, regardless of material or CAM unit. Conversely, the butt joint showed the highest margin trueness, with the bevel line the lowest.
3. When using composite resin, a bevel finish line should be used due to its superior marginal adaptation across both 4‐ and 5‐axis CAM systems; under these conditions, composite resin also outperformed LDS.
4. For lithium disilicate (LDS), the optimal finish line depended on the CAM unit: with a 4‐axis unit, both butt joint and bevel finish lines should be used to achieve better marginal adaptation, with the butt joint providing higher margin trueness, while with a 5‐axis unit, the bevel finish line should be used as it results in the highest marginal adaptation.
5. The 5‐axis CAM unit led to higher margin trueness for LDS with bevel and chamfer finish lines and for CR across all designs, while marginal adaptation was similar between CAM units for bevel and chamfer lines but differed with the butt joint.

## Conflicts of Interest

The authors declare no conflicts of interest.

## Data Availability

The data that support the findings of this study are available from the corresponding author upon reasonable request.
